# The role of cellular reactive oxygen species in cancer chemotherapy

**DOI:** 10.1186/s13046-018-0909-x

**Published:** 2018-11-01

**Authors:** Haotian Yang, Rehan M Villani, Haolu Wang, Matthew J Simpson, Michael S Roberts, Min Tang, Xiaowen Liang

**Affiliations:** 1 Therapeutics Research Group, The University of Queensland Diamantina Institute, The University of Queensland, Translational Research Institute, Level 5 West, Brisbane, Australia; 2 School of Mathematical Sciences, Queensland University of Technology, Brisbane, Australia; 3 Department of Mathematics and Institute of Natural Sciences, Shanghai Jiao Tong University, Shanghai, China; 4 Department of General Surgery, Changzheng Hospital, The Second Military Medical University, Shanghai, China

**Keywords:** Reactive oxygen species (ROS), Redox, Cancer, Chemotherapy, ROS detection, Mathematical modeling

## Abstract

Most chemotherapeutics elevate intracellular levels of reactive oxygen species (ROS), and many can alter redox-homeostasis of cancer cells. It is widely accepted that the anticancer effect of these chemotherapeutics is due to the induction of oxidative stress and ROS-mediated cell injury in cancer. However, various new therapeutic approaches targeting intracellular ROS levels have yielded mixed results. Since it is impossible to quantitatively detect dynamic ROS levels in tumors during and after chemotherapy in clinical settings, it is of increasing interest to apply mathematical modeling techniques to predict ROS levels for understanding complex tumor biology during chemotherapy. This review outlines the current understanding of the role of ROS in cancer cells during carcinogenesis and during chemotherapy, provides a critical analysis of the methods used for quantitative ROS detection and discusses the application of mathematical modeling in predicting treatment responses. Finally, we provide insights on and perspectives for future development of effective therapeutic ROS-inducing anticancer agents or antioxidants for cancer treatment.

## Background

Reactive oxygen species (ROS) is a collective term referring to unstable, reactive, partially reduced oxygen derivatives that are created as a by-product of normal metabolic processes. They include hydrogen peroxide (H_2_O_2_), superoxide anion (O_2_^−^), hypochlorous acid (HOCl), singlet oxygen (^1^O_2_) and hydroxyl radical (·OH), and act as second messengers in cell signaling, and are essential for various biological processes in normal and cancer cells [1]. Many studies have defined ROS as a tumor-promoting or a tumor-suppressing agent, with abundant evidence supporting both arguments [2]. Intracellular balance mechanisms also exist in the form of antioxidant enzymes, major players being Glutathione (GSH) and Thioredoxin (Txn) though a number of antioxidants cooperate to remove ROS species and keep the system in check [3]. Ironically, ROS production is a mechanism shared by most chemotherapeutics due to their implication in triggering cell death, therefore ROS are also considered tumor-suppressing [4]. Recent evidence suggests that prolonged chemotherapy can reduce the overall cellular ROS in cancer, which are believed to function as a key underlying mechanism of drug resistance in chemotherapy [5]. Much of this work has been fueled by a variety of intracellular ROS indicators, from secondary assays to primary observable indicators based on real time fluorescence. It is possible and important to collect this data using effective ROS-detection technology for both the development of models and for the elucidation of biological mechanisms [1]. If robust models were generated, they could form the foundation for future predictions of efficacy, accelerating clinical research outcomes by clearly defining specific redox-dependent vulnerabilities in cancer cells and informing how to avoid global redox changes in normal cells.

In this review, we present evidence about the conflicting roles of ROS as critical secondary messengers in cancer and during cancer chemotherapy. We critically assess current technological advances in quantitative ROS detection that should be more broadly utilized to increase our understanding of redox signaling, and lastly, discuss the application of mathematical modeling in predicting treatment responses and characterizing the signaling pathways induced by chemotherapy-associated ROS.

### The ROS landscape during cancer development

**Fig. 1:**
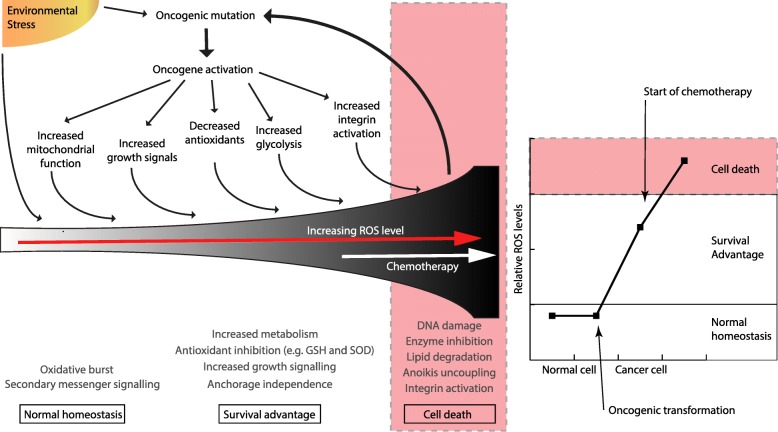
Many factors contribute to increasing ROS levels in cancer, which in turn lead to a number of biological consequences. Overall, current theories suggest the culmination of increased ROS during cancer development confers a survival advantage, which is increased further during chemotherapy. Chemotherapy pushes ROS levels over a critical threshold proposed to induce biological processes leading to cell death, mostly via apoptosis

Normal somatic cells require ROS for a number of cellular processes, such as immune defense mechanisms and obligate secondary signaling [6]. In cancer cells, ROS levels are increased due to both environmental and internal mechanisms (Fig. 1). The overall balance of ROS and the combined positive and deleterious effects of ROS all contribute to the final impact on cancer biology. This topic has been studied extensively in the literature and has been summarized in a number of excellent reviews [7–9]. Firstly, environmental toxins linked to cancer have been shown to increase the amount of ROS species, for example smoking and UV [10, 11]. Also, as ROS are an inevitable by-product of metabolism, the increased metabolism sustaining increased proliferation in cancer cells results in increased ROS production. ROS are generated as a result of activation of a number of well-known oncogenes, for example *Cmyc, Kras* and *BRCA1* [12–15]. ROS are also increased due to hypoxia induced in tumors when the vasculature can no longer adequately supply the growing lesion [16]. Finally, alterations in signaling associated with tumorigenic transformation, such as altered integrin activation during cancer metastasis are also linked to increased ROS species production [17]. All of these mechanisms combined result in a significant increase of cancer cell ROS levels around which there remains much controversy regarding the impact of ROS in the tumor.

In cancer cells ROS are usually considered oncogenic because they have been implicated in initiation, progression and metastasis of cancers however this is not clear cut, as ROS may also be crucial for tumor clearance. A clear mechanism by which ROS impact tumor development is by direct DNA damage during carcinogenic transformation such as catalyzing the modified DNA base 8-OHdG resulting in mutation [18], reviewed by [19]. ROS catalysis of disulfide bond formation can impact a wide range of cellular proteins and lipid modifications which result in unstable, short lived lipids that ultimately propagate reactive species by secondary messenger breakdown products [20]. Finally, anoikis is the process by which normal cells induce apoptosis after the loss of cell matrix attachment. ROS have been shown to promote anoikis resistance and uncouple attachment and programmed cell death in cancer cells, thereby enabling metastasis [21, 22]. While a plethora of information support ROS mediate tumor development, data also supports that ROS removal is correlated with increased tumorigenesis. Antioxidant therapy, which should remove the cancer promoting ROS, paradoxically correlates with decreased survival in clinical trials [23]. This may occur due to antioxidants decreasing ROS to a level supporting tumor proliferation and migration while minimizing some of the negative impacts of ROS in cancer cells, such as DNA damage [24–26]. The obvious contradiction is a continuing area for resolution, and it is becoming more likely that ROS has both positive and negative roles in tumors.

### Cellular sources of ROS during chemotherapy

Most chemotherapeutics generate ROS in cancer cells. It is hypothesized that chemotherapeutic amplification of ROS levels pushes the already increased cancer cells over a threshold to induce cell death (Fig. 1), and is one of the proposed mechanisms by which multiple chemotherapies induce tumor regression [4, 27, 28]. Anthracyclines, such as Doxorubicin, Daunorubicin and Epirubicin, generate the highest levels of cellular ROS [29]. Platinum coordination complexes, alkylating agents, camptothecins, arsenic agents and topoisomerase inhibitors (including epipodophyllotoxin Topoisomerase II inhibitors) also induce high levels of ROS [30–32], while taxanes, vinca alkaloids, nucleotide analogues and antimetabolites, including antifolates and nucleoside, generate lower levels of ROS [4].

**Fig. 2:**
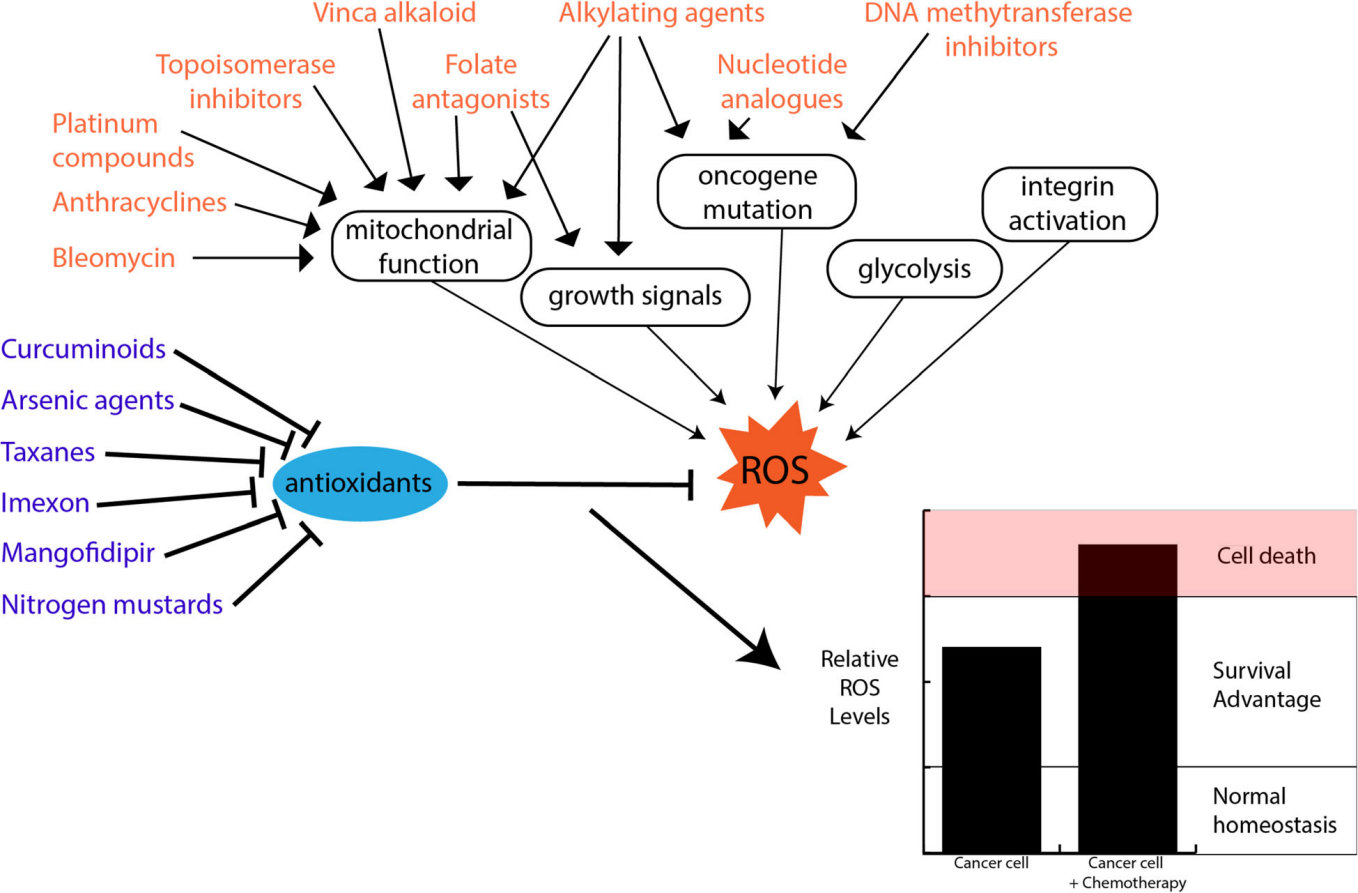
Different chemotherapeutics have distinct mechanisms of action, the diagram represents the cellular mechanisms by which the main classes of chemotherapeutics exhibit their effects. Some chemotherapies, in blue text, impacting ROS production in the cell while others, in orange text, regulate ROS by inhibiting their detoxification by cellular antioxidants. Altered balance of cancer ROS production and removal by chemotherapeutic modulation dictates the final level of ROS and the ultimate outcome of ROS effect

There are two major reasons for elevated cellular ROS production during chemotherapy: mitochondria ROS generation and inhibition of the cellular antioxidant system (Fig. 2). Arsenic trioxide, which was approved for leukemia treatment, has been reported to induce a loss of mitochondrial membrane potential and inhibit complexes I and II, leading to disruption of mitochondrial electron transport chain (ETC) and electronic leakage, and to an elevated ROS production consequently [33, 34]. Many other chemotherapeutics, such as the anthracycline doxorubicin, the antitumor antibiotic Bleomycin, and platinum coordination complexes, also target mitochondria and induce cellular ROS generation [35].

The other major reason for elevated cellular ROS production during chemotherapy is the inhibition of the antioxidant system, which includes low molecular mass antioxidants such as GSH and ascorbic acid, enzymes regenerating the reduced forms of antioxidants, and ROS interacting enzymes such as superoxide dismutase (SOD), peroxidases and catalases [36]. For example, Imexon is a small molecule that binds to thiols such as GSH and cysteine, causing a depletion of cellular GSH and an accumulation of ROS in patients with metastatic cancer [37]. Mangafodipir, a novel adjuvant chemotherapeutic agent, could selectively inhibit SOD in cancer cells and increase cellular H_2_O_2_ levels [38]. For some chemotherapeutics, more than one target site for ROS generation in cancer cells have been defined in experimental and clinical studies. For example, in addition to mitochondrial respiration, the membrane-bound NADPH oxidase (NOX) is another main target of arsenic-induced ROS production [39]. The ROS production by Phenethyl isothiocyanate treatment was reported to involve GSH adducts formation, and inhibition of GSH peroxidase and complex III of the mitochondrial ETC [40].

### Responses of cancer cells to chemotherapy-induced ROS

Many questions regarding the role of ROS in chemotherapy remain, largely focusing on whether the ROS are a major reason for the induction of cell death, or just a side effect induced by the chemotherapy-induced mechanism of cell death. The role of ROS in cellular outcome during chemotherapy is more diverse than anticipated. The cell death triggered by most chemotherapeutics, such as cisplatin, doxorubicin and arsenic agents, involve both ROS-dependent and ROS-independent pathways. For example, the cytotoxic effect of cisplatin, one of the most effective and widely used anticancer chemotherapeutics, is thought to be mediated primarily by the generation of nuclear DNA adducts, which, if not repaired, interfere with DNA replication and cause DNA damage, which can induce cellular ROS generation [41]. However, the ability of cisplatin to induce nuclear DNA damage per se is not sufficient to explain its high degree of effectiveness for the treatment of a number of cancers. Recent work shows that exposure to cisplatin induces a mitochondrial-dependent ROS response that significantly enhances the cytotoxic effect caused by nuclear DNA damage in cancer cells [35]. ROS generation is independent of the amount of cisplatin-induced nuclear DNA damage and occurs in mitochondria as a consequence of protein synthesis impairment.

Cellular responses to chemotherapy-induced ROS reflect the complex integration of ROS type, location, duration, and levels. For example, doxorubicin-induced mitochondrial ROS, particularly H_2_O_2_, are reportedly central to contribute to apoptosis and autophagy in cancer cells [29, 42], while arsenic-induced NOX-generated ROS at the membrane are more often described as contributing cell death via necrosis and ferroptosis [39, 43, 44]. However, these distinctions are not absolute, because membrane-generated ROS can also induce apoptosis [45]. Prolonged exposure to chemotherapy-induced ROS has been reported to induce drug resistance [46]. While implications of ROS in cancer heterogeneity and evolution still lack extensive studies. Chemotherapy may even induce cancer cells to have increased genetic instability due to mutations caused by ROS [47]. The dynamic sequence of some chemotherapy for cell readjustments may eventually promote the evolution of resilient and drug-resistant cells, which can repopulate the tumor and contribute to the emergence of a new heterogenic, more metastatic and drug-resistant tumor [5]. Although it is questionable if mitochondrial ROS are important contributors to drug resistance, its role and modulation of metabolic events may be central to the process and results [1].

## Methods for quantitative ROS detection

As critical secondary messengers in the cell, ROS involvement in cancer chemotherapy is not confined to indiscriminate macromolecular damage. It is both topological and temporal, and ROS-dependent signaling is expected to be regulated in a time- and space-dependent manner. Thus, quantitative monitoring of the activity of ROS with appropriate spatiotemporal resolution is essential to defining the source and kinetics of redox signaling, which will be fundamental to resolving the ROS conundrum. Currently while there are many approaches to quantitatively monitoring of ROS activity, none of these technologies have reached a standard enabling clinical ROS detection and these technologies therefore will need to be developed further to enable clinical use (Table 1) [48, 49].

**Table 1:** Methods and developments in ROS detection

	Advantages	Disadvantages	References
ROS detection method			
Secondary oxidation product detection	Minimally invasive; Clinically used currently; Quantification feasible	Cannot visualize spatio-temporal ROS	[60]
Small molecule colorimetric assays	Simple chemistry; Quantification feasible	Cannot visualize ROS in real time	[42]
Redox sensitive fluorescent small molecules	High sensitivity; High spatial resolution (subcellular levels); Less expensive; Detect specific ROS types; Ex vivo histological detection possible	Drawbacks with stability and imaging time; Cytotoxicity of certain probes; Not good for longitudinal studies	[61, 62]
Redox sensitive Fluorescent proteins	Tracking over unlimited time (built-in probes); Allows whole-body scanning; Targeted localization (subcellular levels)	Genetic modifications of cells/animals required	[49–51]
Recent technological optimization			
FLIM and FRET based probes	Increased specificity and sensitivity; Multimodal imaging capability; High sensitivity; High spatial resolution (molecular levels)	More complex probe construction; Costly equipment	[43, 44]
Nanoparticle delivery systems	Capacity for multiple cargos; Increased specificity and sensitivity; Enable targeted probe delivery	More complex probe construction	[63]

*Abbreviations: FRET* Fluorescence resonance energy transfer, *FLIM* fluorescence-lifetime imaging

**Fig. 3:**
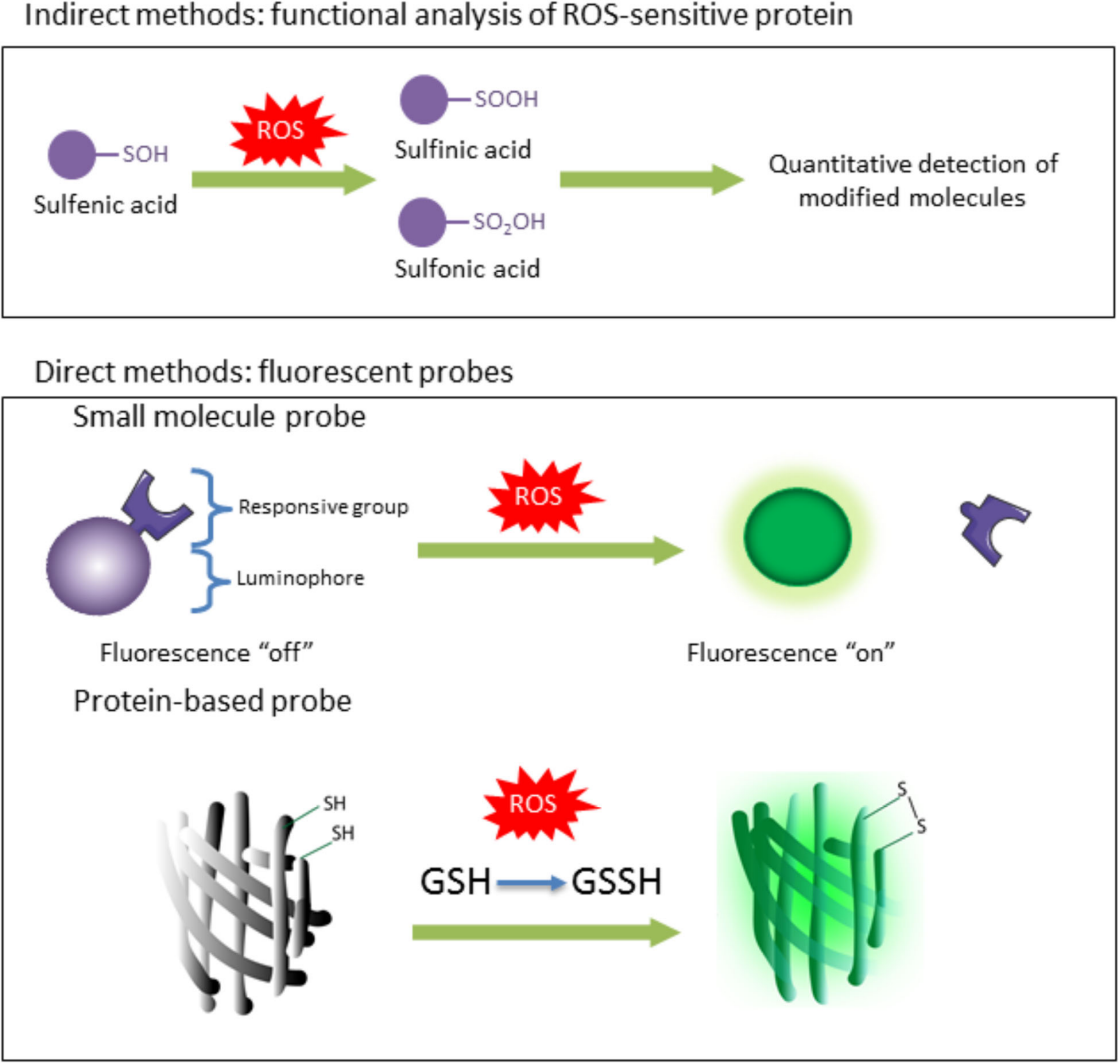
ROS detection has been performed using a variety of different methods. Indirect analysis of ROS is performed by the analysis of the oxidation products of ROS. More direct methods of ROS analysis include the visualisation of small molecules that convert to an alternative spectrum of fluorescence after ROS mediated oxidation. Protein based probes function with a similar theory, the ROS mediated oxidation of residues in the fluorescent protein alters the emission of the protein enabling localisation of ROS oxidation

Conventional ROS detection methods, such as chemical and immunological approaches, have been well developed for functional analysis of cellular ROS-sensitive proteins in biopsies, cell lines or harvested tissues during different stages of chemotherapy using direct or indirect methods for ROS detection (Fig. 3). For instance, using these methods, protein sulfenic acid modifications, oxidative cysteine modifications and unrelated sulfinic or sulfonic acid modifications can be directly detected, which constitute the main regulatory target of ROS [50, 51]. Early versions of these technologies relied upon alterations in changes in electron density or enzymatic -based colorimetric changes, meaning visualization was limited to fixed and static detection methods [52]. Since then, fluorescent probes for ROS-detection have been developed to track the dynamics of specific ROS in real time. These probes usually integrate a specific responsive group for ROS with suitable luminophores, such as fluorescein, rhodamine, coumarin, cyanine, and metal complexes [53, 54]. These small molecule based indicators in general detect ROS through the same mechanisms as the conventional ROS detection mechanisms, but emit fluorescent signals after sulfenic acid modifications, oxidative cysteine modifications and unrelated sulfinic or sulfonic acid modifications [55, 56]. Small molecule ROS probes have also been optimized to increase their sensitivity and specificity. For example, metal complex-based probes are suitable for multi-signal detection and multi-modal imaging, excluding the influence by fluid optical properties, endogenous fluorophores, probe concentration, and other in vivo environmental or instrumental factors [53, 57, 58]. Finally, development of bioluminescent probes have enabled non-invasive in vivo imaging capabilities which provide a tantalizing opportunity for detecting dynamics of ROS in patients [59].

Protein based probes have also been developed based on fluorescent proteins modified for redox sensitivity, the main benefits being these probes can be genetically-encoded such that they are targeted to specific cellular compartments to detect any spatiotemporal ROS changes [60, 61]. The fluorescent protein-based redox probes that have been developed are now providing, for the first time, an opportunity to visualize and quantify the long-term ROS fluctuation in live cells [62]. Finally, regardless of small molecule or protein technology, these methods, when used in combination with advanced imaging techniques, such as multiphoton intravital imaging and in combination with florescent technologies such as fluorescence resonance energy transfer (FRET) and fluorescence-lifetime imaging (FLIM), increased sensitivity and specific localization has also been achieved [63]. The high sensitivity and more diverse imaging ability enabled by such probes widens the applicability of such compounds and represent a new direction for ROS study.

The chemotherapy-induced ROS detection in clinical settings has been inferred by the elevation of lipid peroxidation products and the reduction antioxidants such as GSH, vitamin E, vitamin C and β-carotene in blood plasma [4]. Despite the significant developments in ROS detection, there is no real-time direct ROS method for human clinical use. Magnetic resonance techniques such as electron paramagnetic resonance (EPR) and magnetic resonance imaging (MRI) have high potential as clinically viable ROS detection methods, these techniques detect endogenous nitroxides, though a probe is required to facilitate detection. Hydroxylamine or acetoxylamine probes are most clinically viable probe option for EPR, due to their low toxicity and relative stability, however they remain to be applied in the clinic as the depth of imaging is not enough for human clinical study [38, 64–66]. Therefore, the challenge for cancer biology remains to develop clinical methods to detect ROS in cancer in a spatiotemporal manner in vivo, within the human body. This would help to resolve some of the previously mentioned contradictions and enable prediction of developing therapeutics in the complex in vivo situation.

In particular, the latest generation of these fluorescent molecular probes are becoming increasingly attractive due to their inherent advantages such as high sensitivity and specificity, rapid analysis, and easy management. These biochemical tools provide a facile platform to interrogate the differences in ROS in normal versus cancer cells. This begins the identification of cancer-specific redox dependencies that may be therapeutically actionable. However, the bottlenecks of these molecular probes are difficulties in tracking dynamic ROS behavior because of their short half-life and their low targeting efficiency. These experimental approaches will undoubtedly open the door to novel cancer biology.

### Mathematical modeling of the chemotherapy-associated ROS

**Fig. 4:**
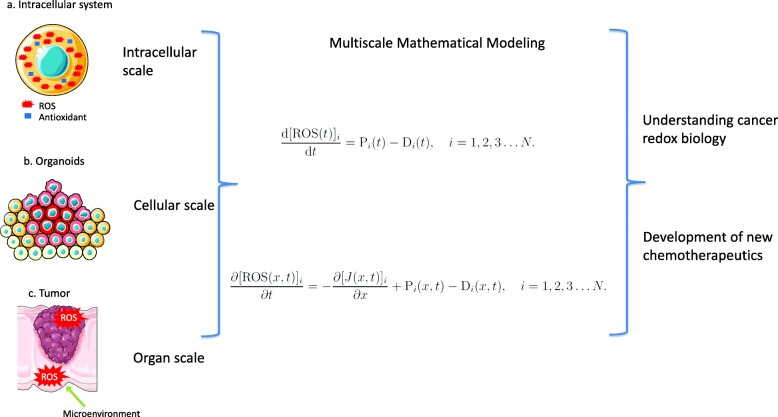
Schematic representation of the mathematical modeling of cancer at an intracellular, cellular and organ scale. Because tumors are heterogeneous entities in a changing microenvironment, development of new chemotherapeutics and understanding the sophisticated cancer redox biology are needed to address the importance of diversity in cancer cell populations and microenvironmental characteristics. Integrating information from multiple levels of biological complexity and multiscale models can potentially be more powerful than focusing solely on the well-developed molecular network level. In this framework, a system of ordinary differential equations could be developed to describe the dynamics of N species, [ROS]1(t), [ROS]2(t), [ROS]3(t) …[ROS]N(t), where the dynamics are governed by the production and decay terms for each ROS species, Pi(t) and Di(t), for *i* = 1,2,3…N, and t is time. In addition, each ROS species varies both temporally and spatially, such as at the organ-scale, it would be more appropriate to work with a system of partial differential equations. For this situation the mathematical model would predict the spatiotemporal distribution of N species, [ROS]1(x, t), [ROS]2(x,t), [ROS]3(x,t) …[ROS]N(x,t), where t is time and x is spatial position. In this case the spatial transport of each ROS species is governed by the flux J(x,t), which could be used to specify diffusive transport or some kind of directed transport if appropriate

Mathematical modeling is an important tool that can provide a robust framework to better understand cancer progression, predict responses to chemotherapy, and to optimize drug dosing protocols. The essential mechanisms for tumor progression are usually buried in overwhelmingly complex physiological details and involve multiple space and time scales [67, 68]. Mathematical modeling of cancer is dissected at different scales including: systems for intracellular pathways; population models describing the tumor cell expansion; systems studying the tumor-microenvironment interactions and models at the whole human organ level (Fig. 4) [69, 70]. Despite ROS playing a crucial role in cancer biology, publications on mathematical modeling and analysis are still limited and multiscale mathematical modeling of ROS in cancer is at a very early stage. In this review, we focus on models with ROS involvement in cancer chemotherapy.

Different kinds of continuum mathematical models are relevant in different situations. For example, to develop a mathematical model to describe intercellular dynamics of ROS might be sufficient to treat the intercellular environment as being well-mixed so that each ROS species depends only upon time. In this framework, a system of ordinary differential equations (Fig. 4) could be developed to describe the dynamics of *N* species, [ROS]_1_(t), [ROS]_2_(t), [ROS]_3_(t) …[ROS]_*N*_(t), where the dynamics are governed by the production and decay terms for each ROS species, P_i_(*t*) and D_i_(*t*), for *i* = 1,2,3…*N*, and *t* is time. To apply this kind of model one must first decide upon how many ROS species are relevant to the application of interest, and what those ROS species are. Furthermore, we must define how the production and decay terms are defined so that they represent the key chemical and biochemical reactions that govern the dynamics of each ROS species thought to be relevant. Of course, in this kind of formulation we must apply certain assumptions, such as making decisions about which ROS species are present and relevant. However, the strength of using a mathematical modeling framework is that these assumptions can be easily revisited and revised to examine how those assumptions impact the prediction of the mathematical model. This process can be particularly powerful in elucidating biological phenomena when the predictions of a mathematical model are tested using experimental observations, giving rise to an iterative predict-refine-predict process.

In the case where it is thought that each ROS species varies both temporally and spatially, such as at the organ-scale, it would be more appropriate to work with a system of partial differential equations (Fig. 4**)**. For this situation the mathematical model would predict the spatiotemporal distribution of *N* species, [ROS]_1_(*x,t*), [ROS]_2_(*x,t*), [ROS]_3_(*x,t*) …[ROS]_N_(*x,t*), where *t* is time and *x* is spatial position. In this case the spatial transport of each ROS species is governed by the flux, *J(x,t)*, which could be used to specify diffusive transport or some kind of directed transport if appropriate [71]. Again, in the partial differential equation framework the local dynamics of each ROS species are governed by the production and decay terms, P_i_(*x,t*) and D_i_(*x,t*), for *i* = 1,2,3…*N*. Using this kind of differential equation description, it would be possible to test different hypothesis about how different species of ROS affect various cellular-level functions, such as cell proliferation or cell death, by coupling the mathematical model of ROS dynamics to a model of cellular behavior [72].

In early studies of cancer redox biology, models focused on specific biochemical pathways to provide potential therapeutic targets. For example, Qutub et al. presented a model for the intracellular pathways that explains how ROS and antioxidants affect the HIF1 pathway in cancer [73]. It was used to explore how combined doses of potentially therapeutic targets (iron, ascorbate, hydrogen peroxide, 2-oxoglutarate, and succinate) affects the expression of HIF1. This kind of model includes multiple feedbacks due to ROS-driven signaling, and intuitive reasoning is insufficient to understand the whole dynamics. Recently, cell population level models that consider tumor-microenvironment interactions were proposed to examine the efficacy of chemotherapy [74, 75]. By specifying the initial tumor size and initial biochemical conditions (e.g. oxygen concentration, pH, glutathione, and redox conditions), these models can predict the time- and space-dependent tumor growth during, and after chemotherapy [75]. They allow preclinical studies on chemotherapy-associated ROS in animals to semi-quantitatively translate into humans, and are used to test in silico different therapeutic protocols. We anticipate that these theoretical framework mathematical models for ROS will lead to second-generation multiscale models incorporating data from the aforementioned novel quantitative ROS detection methods to address the role of diversity in cancer cell populations and the organ microenvironment (Fig. 4). By integrating information from multiple levels of biological complexity, these advanced models can potentially be more powerful than focusing solely on the well-developed molecular network level in improving understanding of the sophisticated workings of redox biology in cancer and guiding the development of new chemotherapeutics.

## Conclusions and prospects

As outlined above, ROS are of indisputable importance in cancer chemotherapy. ROS do not serve as simple biochemical entities, but as topological and temporal secondary messengers in cancer cells. Although most chemotherapeutics globally increase ROS to cytotoxic levels in targeting cancer cells, such ROS exposure may also inevitably reduce the efficacy of chemotherapy in the long term. To leverage cellular redox changes towards the development of a safe and effective therapeutic strategy necessitates experimental delineation of specific redox signaling pathways that are uniquely required by cancer cells to grow, survive or die. In this regard, our understanding of the complicated redox biology in cancer is still in its infancy. We anticipate that new delivery strategies, such as nanoparticle delivery systems, will be developed and applied in the clinic to further increase cellular ROS levels in cancer and reverse drug resistance. New chemotherapeutics can be engineered to target to specific cellular compartments for ROS generation and maintenance for a certain period of time.

ROS-detection fluorescent probes with temporal and spatial specificity have illuminated the diverse nature of ROS-mediated cell signaling events, and will shed further light on the relationship between different redox couples and how they operate in different cellular compartments. Further elucidation of the functional consequences of ROS using mathematical models will be crucial to advancing our understanding of complex diseases, especially cancer. A multidisciplinary collaboration between experimental, modeling and clinical areas will be required to integrate modern mathematical modeling together with the experimental techniques and the expertise needed for ROS detecting, analyzing and clinical translation. More second-generation models will be developed to improve the understanding of the sophisticated workings of cancer redox biology, and to propose designs of new chemotherapeutics to defeat cancer.

## Abbreviations

EPR: Electron Paramagnetic Resonance
ETC: Electron Transport Chain
FLIM: Fluorescence-Lifetime Imaging
FRET: Fluorescence Resonance Energy Transfer
GSH: Glutathione
MRI: Magnetic Resonance Imaging
NOX: NADPH Oxidase
ROS: Reactive Oxygen Species
SOD: Superoxide Dismutase
Txn: Thioreduoxin
